# Genome Sequence of a Clinical Blood Isolate of Kodamaea ohmeri

**DOI:** 10.1128/mra.00843-22

**Published:** 2022-11-15

**Authors:** Ka Lip Chew, Rosemini Achik, Nurul Hudaa Osman, Sophie Octavia, Jeanette W. P. Teo

**Affiliations:** a Department of Laboratory Medicine, National University Hospital, Singapore; b Environmental Health Institute, National Environment Agency, Singapore; Vanderbilt University

## Abstract

Kodamaea ohmeri is a rarely occurring yeast that can cause human infections. We describe the whole-genome sequence of a K. ohmeri clinical blood isolate.

## ANNOUNCEMENT

**FIG 1:**
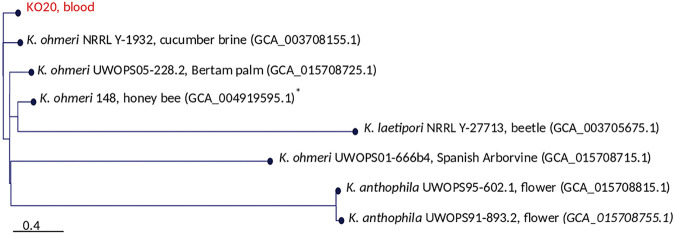
Core SNP tree for KO20 (this study), other publicly available Kodamaea ohmeri genomes, and other *Kodamaea* species genomes. The scale bar denotes the number of substitutions per site. The strain name, isolation source, and GenBank assembly accession number (in parentheses) are provided for each strain. *, Representative K. ohmeri genome.

Kodamaea ohmeri, formerly Pichia ohmeri, is a teleomorph of Candida guilliermondii var. *membranifaciens*. It is a rarely occurring yeast that is usually isolated from environmental settings, such as pools and seawater (1), but has been known to cause fungemia (2) and hospital outbreaks (3, 4). Here, we describe the whole-genome sequence of a clinical blood isolate of K. ohmeri (named KO20). The other publicly available genomes were from environmental sources, including the representative genome, strain 148, from honeybees (5) (Fig. 1).

In 2020, at a local hospital (Singapore), a positive blood culture bottle was subcultured onto chocolate blood agar and MacConkey agar, as well as CHROMagar *Candida* medium (Becton Dickinson) and Sabouraud dextrose agar (SAB), since yeast colonies were observed. Matrix-assisted laser desorption ionization–time of flight mass spectrometry (MALDI-TOF MS) using Bruker Biotyper (Bruker Daltonik GmbH, Bremen, Germany) and Vitek MS (bioMérieux, Marcy-l'Étoile) systems identified the isolate (KO20) as K. ohmeri from SAB plates. The patient was a 25-year-old female patient with myelodysplastic syndrome who underwent stem cell transplantation. The patient was treated initially with liposomal amphotericin B and then intravenous voriconazole, which led to an uneventful recovery.

DNA was prepared from overnight cultures in liquid yeast extract-peptone-dextrose (YPD) medium by using the DNeasy blood and tissue kit (Qiagen) and following the manufacturer’s yeast protocol. The DNA library was prepared using the Illumina Nextera DNA Flex library preparation kit and sequenced with an Illumina HiSeq system, which generated 150-bp paired-end reads. A total of 8,057,053 reads were obtained. Low-quality bases and adapters were removed using Trimmomatic v0.38 (6) and assembled using Shovill v1.0.4 (https://github.com/tseemann/shovill). QUAST v5.0.2 was used to evaluate the quality of the genome assembly (7). Assessment of the genome completeness was performed using bench marking universal single-copy orthologs (BUSCO) with the saccharomycetes data set (8). Whole-genome alignment of the *de novo* assembled contigs with other *Kodamaea* species whose genomes are publicly available (GenBank accession numbers JANDME000000000.1, GCA_004919595.1, GCA_003708155.1, GCA_015708715.1, and GCA_015708725.1) was performed using a k-mer-based method (9), and the core genome tree was constructed using FastTree (10) (Fig. 1).

The average depth of coverage was 92.5×. The K020 assembly aligned to 98% of the representative K. ohmeri strain 148. A total of 2,137 BUSCO groups were searched, and 2,104 complete BUSCOs were detected, with a completeness score of 98.4%. The assembly statistics (Table 1) reflect very similar genome sizes and GC contents of 12.4 Mb and 42.7%, respectively, compared to other K. ohmeri genomes. Phylogenetic relationships showed that the single-nucleotide polymorphism (SNP) distances between KO20 and the four other available K. ohmeri genomes ranged between 30,835 and 103,533 (Fig. 1).

**TABLE 1:** Assembly statistics for KO20 in comparison with other publicly available Kodamaea ohmeri genomes

Strain	GenBank accession no.	Source	Genome size	No. of scaffolds	Contig *N*_50_	Contig *L*_50_	GC content (%)
KO20 (this study)	JANDME000000000.1	Blood (fungemia)	12,353,342 bp	105	259,155 bp	16	42.70
148^*a*^	GCA_004919595.1	Honeybee midgut	12.6 Mb	20	1.8 Mb	3	42.5
NRRL Y-1932	GCA_003708155.1	Cucumber brine	12.4 Mb	118	302.4 kb	11	42.5
UWOPS01-666b4	GCA_015708715.1	Distimake tuberosus (Spanish arborvine)	12.3 Mb	62	1.4 Mb	15	42.5
UWOPS05-228.2	GCA_015708725.1	Bertam palm	12.3 Mb	89	580.2 kb	8	42.5

a Representative genome in GenBank.

This project was reviewed and approved by the National Healthcare Group Domain-Specific Review Board (reference number 2020/01431).

### Data availability.

This whole-genome shotgun project has been deposited in DDBJ/ENA/GenBank under the accession number JANDME000000000.1. Raw sequence reads have been deposited in the NCBI Sequence Read Archive (SRA) under accession number SRP386360.
